# Energy Harvesting from the Stray Electromagnetic Field around the Electrical Power Cable for Smart Grid Applications

**DOI:** 10.1155/2016/3934289

**Published:** 2016-08-07

**Authors:** Farid Ullah Khan

**Affiliations:** Institute of Mechatronics Engineering, University of Engineering & Technology Peshawar, Peshawar 25000, Pakistan

## Abstract

For wireless sensor node (WSN) applications, this paper presents the harvesting of energy from the stray electromagnetic field around an electrical power line. Inductive and capacitive types of electrodynamic energy harvesters are developed and reported. For the produced energy harvesters, solid core and split-core designs are adopted. The inductive energy harvester comprises a copper wound coil which is produced on a mild steel core. However, the capacitive prototypes comprise parallel, annular discs separated by Teflon spacers. Moreover, for the inductive energy harvesters' wound coil and core, the parametric analysis is also performed. A Teflon housing is incorporated to protect the energy harvester prototypes from the harsh environmental conditions. Among the inductive energy harvesters, prototype-5 has performed better than the other harvesters and produces a maximum rms voltage of 908 mV at the current level of 155 A in the power line. However, at the same current flow, the capacitive energy harvesters produce a maximum rms voltage of 180 mV. The alternating output of the prototype-5 is rectified, and a super capacitor (1 F, 5.5 V) and rechargeable battery (Nickel-Cadmium, 3.8 V) are charged with it. Moreover, with the utilization of a prototype-5, a self-powered wireless temperature sensing and monitoring system for an electrical transformer is also developed and successfully implemented.

## 1. Introduction

The increasing interest in abandoned, embedded, and implantable wireless sensor nodes (WSNs) in remote, harsh, inaccessible, and hazardous locations has greatly influenced the requirement of alternative energy source for the batteries used for their operation. The limited life of batteries in these WSNs restricts the applications of WSNs to a very small domain. Moreover, the charging and replacement of these batteries are also not feasible in applications where thousands of such WSNs are operating in a wireless network which is spread over a vast area. Furthermore, these batteries are environmental hazard and require a standard procedure for disposal. The integration of WSN with the alternative and renewable energy source, such as energy harvester, will transform a traditional WSN into an autonomous or self-powered WSN. The energy harvester will convert the energy that is present in its surroundings into the electrical energy which will be utilized for the operation of a WSN. Such an autonomous WSN will not require any charging and replacement and can be operational for much longer times at locations where the traditional WSN is not applicable and feasible.

With WSNs, the sensing and monitoring of high voltage transmission lines [1, 2] are gaining a sharp interest in recent times. Moreover, the temperature monitoring of smart electricity grids [3, 4] is also performed with wireless temperature sensor nodes (WTSNs) [5–7]. With the WTSNs, the temperature of the critical spots within the electrical transformers or feeders is continuously monitored to safeguard these devices and ensure no power outages. Some commercially available battery power WTSNs are listed in Table 1. These WTSNs can be utilized to monitor the temperature from −55 to 150°C. For the tabulated WTSNs, the data transmission range is from 10 to 90 m, except in WTSN module [8], where GSM technology is employed to monitor the temperature. Due to the application of Arduino microcontroller units in these WTSNs, the operational voltage is more or less equal and ranges from 4 to 6 V. During sleep mode, functioning very small amount of energy is consumed by WTSNs; however, in data transmission operation, a current consumption is from 30 to 1300 mA. In the transmission mode the overall power utilization in the commercial WTSNs is actually from 120 to 7800 mW.

Energy harvesting technology developed for powering the WSNs is quite capable of operating the WTSNs in electrical transformers. For energy harvesters, numerous energies are available in the environment of an electrical transformer, such as vibration, acoustic, solar, and thermal. These energies can be extracted with the developed vibration-based [13–15], acoustic-based [16, 17], solar-based [18, 19], and thermal-based [20, 21] energy harvesters. Moreover, stray electromagnetic field is also abundantly present around the power cables of the transformers. This alternating stray electromagnetic field can be utilized to produce enough power for the operation of WTSNs mounted for the temperature monitoring of the electrical transformers.

For wireless sensors, in a smart grid an energy harvester is developed to harvest a stray electric field around an electric power cable [22]. The reported energy harvester consisted of a 60 cm long aluminum foil (2 mm thick) that is wrapped on top of the insulated power cable (7 mm diameter) and a storage capacitor which is connected to the aluminum foil through the charging circuit. The diameter of the conducting wires inside the power cable is about 1.5 mm. When a voltage of 220 V is supplied to the power cable, between the harvester's aluminum foil and inner conducting wire of the cable, a capacitance of 6.8 *μ*F is obtained. This capacitance is utilized to generate energy. It is reported that the generated capacitance in the energy harvester is directly related to the foil's length. When an aluminum foil of length 20 cm is wrapped around the cable and a storage capacitor (2 *μ*F) is integrated to the energy harvester, an average current level of 1570 nA is obtained from the setup. However, when the length of aluminum foil is increased to 60 cm, an average energy of 2 mJ is reported to be collected at the storage capacitor (47 *μ*F) in only 35 sec. The current flow to the storage capacitor is about 4.53 *μ*A and an average power generation of 47 *μ*W is reported for the developed energy harvester. Moreover, when the reported harvester is connected to a WSN, the WSN successfully transmitted the measured data every 42 sec. A stray alternating electromagnetic field surrounding the power cable is exploited to operate a piezoelectric type energy harvester for the generation of power in [23]. The reported energy harvester comprised a piezoelectric bimorph (Piezo Systems Inc., T215-A4SS-103X) cantilever beam (31.8 × 3.2 × 0.38 mm) and two neodymium (NdFeB) strong disc magnets which are mounted at the free end of the piezoelectric cantilever beam. Aluminum brackets are utilized to clamp one end of the beam. The developed energy harvester is then mounted over the power cable which is delivering power to a 1500 W heater. Due to the interaction of the cable's alternating stray magnetic field and the field of a permanent magnet at the free tip of the piezoelectric beam, the beam oscillates. For optimal operation, the resonant frequency (60 Hz) of the device is matched to the frequency of current flowing in the power cable. When the optimum load resistance (491 kΩ) is attached to the harvester, it produced a maximum power of 345 *μ*W for a cable current level of 13 A. Moreover, a full-wave bridge rectifier is utilized for the conversion of output AC voltage of the harvester into the DC voltage and a capacitor from 0 to 5 volts is reported to be charged with the energy harvester in 196 sec. A hybrid type of energy harvester developed for the monitoring of an electrical transformer [24] consisted of solar-type and vibration-type energy harvesting elements. The energy harvester is developed to simultaneously extract the energy from the vibration of an electrical transformer and the ambient solar energy available near the transformer. The vibration-based energy harvesting portion consisted of a bimorph piezoelectric beam (PCFC-100, Advanced Cerametrics Inc.) that is clamped at the mid span to form two attached cantilever beams. Proof masses (copper, 3 gram each) are mounted at the free ends of these beams. For optimum power generation the device resonant frequency is kept around 100 Hz. The cantilever beam setup is kept enclosed in a casing. The solar energy harvesting portion of the prototype consisted of a flexible solar panel (MP3-37, 114 × 37 × 0.2 mm,) which is placed on top of the casing. For the vibration portion of the hybrid energy harvester, a maximum power generation of 897 *μ*W is reported when the prototype is connected to 300 kΩ (optimal load resistance). Moreover, the voltage obtained from the piezoelectric beams is rectified to a DC voltage and, only in 38 sec, a 1000 *μ*F capacitor is then charged from a voltage level of 400 mV to 5.12 V. On the other hand, from the solar energy harvesting portion of the device, a maximum voltage (open circuit) of 3.95 V is generated and for it a maximum power of 31.43 mW is reported to deliver a load resistance of 301.6 Ω. Furthermore, the solar energy harvester is used to charge a super capacitor (1 F) from 720 mV to 3.5 V in 7 minutes. An energy harvester [25] developed for generating energy from the stray electromagnetic field around the transmission line comprised an open core (x-shaped, 54 × 64 × 18 mm) over which an enameled copper wire is wound around to produce a 300-turn coil. The reported electromagnetic energy harvester is attached to the transmission line with the help of a plastic zipper band. For a current level of 800 A in the transmission cable, the energy harvester generated a maximum power of 90 mW. However, an alternating voltage (open circuit) of 200 mV is reported for the energy harvester under a cable current level of 60 A. Furthermore, a rectifier is utilized to transform the AC output of the harvester to DC which is then delivered to charge a storage capacitor for WSN applications. For wireless sensor applications in electrical power systems, an electrodynamic energy harvester [26] is produced that is composed of a five-layered cylindrical core made from a flexible magnetic material (mu-metal) and a wound coil (280 turns) produced by wrapping a copper wire over the core. When the energy harvester is mounted over the power cable, a voltage amplitude of 0.88 V is obtained for a cable current level of 13.5 A. Moreover, under the same current level and being connected to the optimum impedance of 76.1 Ω, an optimum power of 10.385 mW is reported for the harvester. However, with a multiplier circuit, the AC voltage from the harvester is stepped up to a DC voltage (2.4 V) which is then utilized to completely charge a 1.2 V rechargeable battery in 3 hours. For a stick-on wireless temperature and current sensor, an electrodynamic energy harvester is developed by [27]. The reported harvester is made of an x-shaped steel laminated core, over which 28 AWG enameled copper wire is wrapped to form a 200-turn wound coil. Both the sensor kit and energy harvester are clamped to the power transmission line. For the energy harvester, a voltage (open circuit) generation of 0.2 V is reported for a cable current level of 50 A. However, after incorporation of a novel AC to DC boost-converter circuit, the energy harvester-circuit unit provided a stable 3.3 V output voltage for a wide cable current level that ranged from 60 to 1000 A. Furthermore, the energy harvester is also used to charge a backup 1 F ultracapacitor in order to operate the sensors in case of power outage. An electrodynamic energy harvester [28] reported for a WSNs application comprised a mu-metal core and a copper wound coil. Rectangular pieces (each 0.014 in thick) of mu-metal are bonded together to produce the harvester's core and a magnetic wire (30–34-gauge) is used to make a wound coil of 500 turns. For a current level of 12 A, the energy harvester produced an rms voltage of 470 mV and a maximum power of 7 mW under a matched impedance of 25 Ω. Moreover, with a Cockcroft-Walton AC to DC step-up converter, a voltage rectification up to 15 V DC (open circuit) is obtained from the developed energy harvester. For the monitoring of AC power supply lines, a self-powered WSN is developed in [29]. The reported self-powered system comprised a temperature sensor, a current sensor, an energy harvester, and a wireless transmission unit. The device is composed of a split-core that has two sets of wound coils, one is used for the measurement of cable current and the other is being utilized to harvest the energy from the stray magnetic field around the power line. An on-board power management circuitry is used to rectify the output AC voltage of the harvester into a DC voltage for the sensor and wireless transmission unit operations. The developed system is reported for monitoring of a current level (from 1 to 100 A) in an AC power line attached to electrical appliances. A self-powered WSN [30] developed for temperature monitoring of overhead power transmission lines is composed of an electromechanical energy harvester, temperature sensors, power conditioning circuit, and wireless transmission module. The energy harvester reported in this work is piezoelectric bimorph cantilever beam having two permanent magnets at the free end. The interaction between the field of the permanent magnets and the changing magnetic field around the power line causes the beam to oscillate and produce power. In order to avoid the failure of the harvester due to large faulty current surges, mechanical stopper is provided in the setup. For the devised harvester a power generation of 100 *μ*W to 1 mW is reported for a current level from 30 to 142 A, respectively.

This work reports on a fabrication and characterization of electrodynamic energy harvesters developed for the stray magnetic field surrounding the output transmission cable of an electrical transformer. In order to envision the magnetic field inside and outside the power cable, a 2-dimensional magnetostatic analysis is conducted in COMSOL Multiphysics for a single-core as well as for a three-core power cable. Based on the simulations in COMSOL Multiphysics, a number of inductive and capacitive energy harvester prototypes are produced and characterized at various levels of electric current flowing in the transformer's output cable. The AC output produced by the energy harvester is converted into DC voltage and is used to charge a super capacitor and a rechargeable battery, which are then utilized to successfully operate a WTSN mounted on an electrical transformer for temperature monitoring.

## 2. Harvester's Architecture and Operational Mechanism

**Figure 1:**
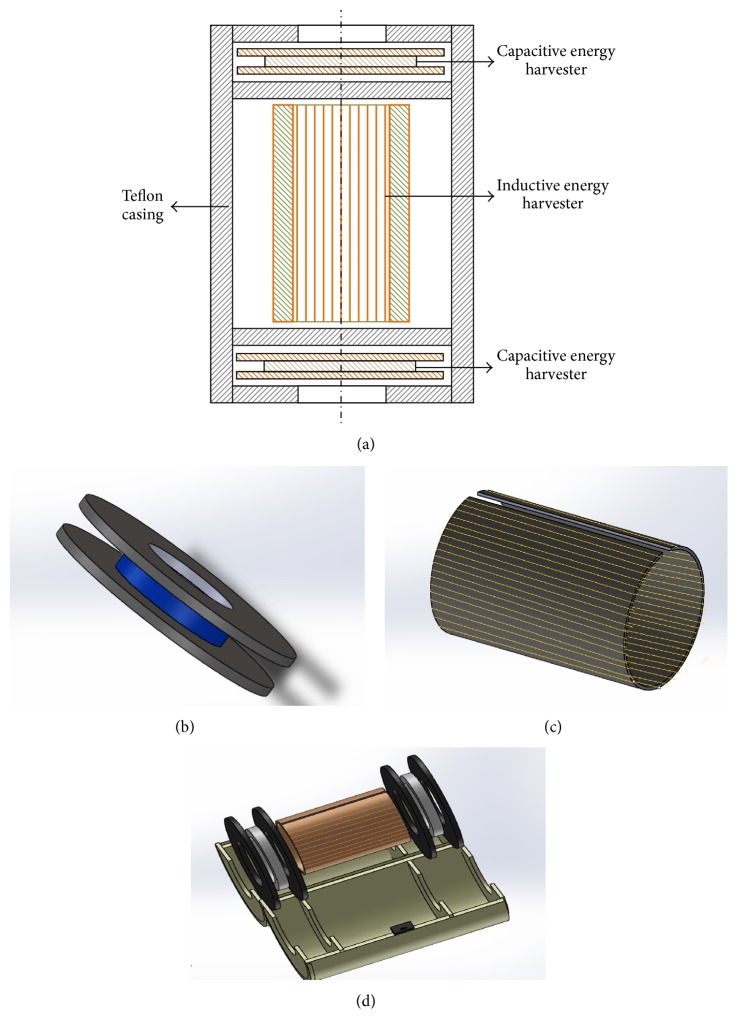
Schematic of the electrodynamic energy harvester: (a) cross-sectional view of the harvester, (b) schematic of the harvester's capacitive unit, (c) schematic of the harvester's inductive unit, and (d) exploded view of the harvester with the Teflon casing.

The hybrid energy harvester developed in this work is shown in Figure 1. The developed harvester is composed of two capacitive and one inductive energy harvesting unit.  Figure 1(a) shows the cross-sectional view of the device having an inductive harvester located in the middle and capacitive harvesters residing at both ends. These harvesters are placed in a split cylindrical casing. In the prototype, the capacitive harvester is made up of two parallel metallic plates with a Teflon ring used as a spacer in between (Figure 1(b)). Moreover, a hole is provided in the center of these disc plates for passing the power cable through them. However, the inductive harvesting unit comprises a steel sheet core over which an enameled copper wire is wrapped longitudinally to develop a wound coil (Figure 1(c)). The core of the inductive harvester is made cylindrical in shape for allowing the power cable to pass inside the core.  Figure 1(d) shows the exploded view of the harvester assembly in the Teflon casing. The function of the casing is not only to keep the three energy harvesting units apart and over the power cable but also to safeguard these units from the environmental hazards, such as rain and scorching heat.

When the energy harvester is installed around the power line, the alternating current in the line causes a changing stray electromagnetic field surrounding the power line. In the inductive energy harvesting unit, due to the alternating stray magnetic field, an emf will be induced in the individual turn of the wound coil according to Faraday's law of electromagnetism. However, this alternating magnetic field in between the parallel plates of the harvester's capacitive unit will induce an alternating electric field across the plates that is responsible for the generation of voltage across the conductive plates.

## 3. Modeling and Simulation

### 3.1. Finite Element Analysis of a Power Cable

**Figure 2:**
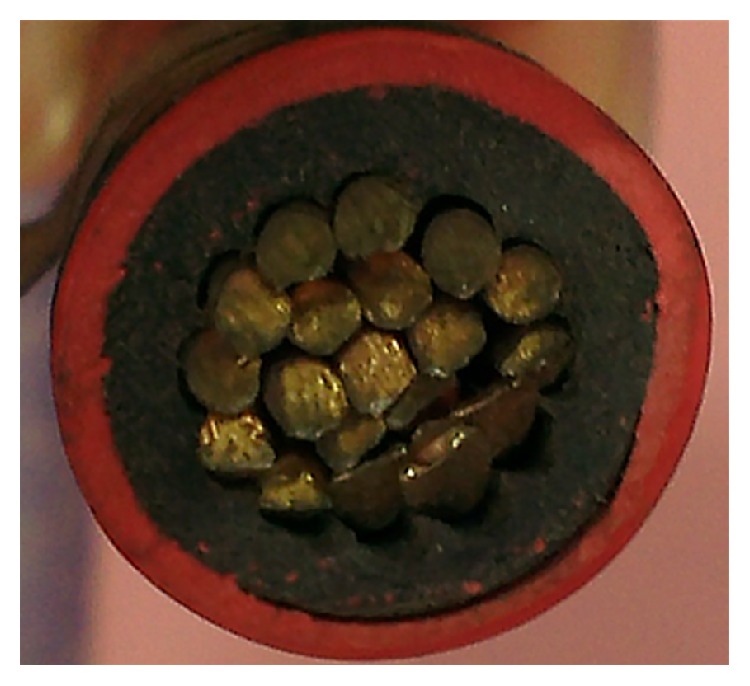
Single-core insulated cable for electrical power transmission.

Simulation of magnetic flux density (MFD) around an output electrical cable of an electrical transformer is performed in COMSOL Multiphysics. Normally, two types of insulated cables (single-core and three-core) are used to connect electrical transformers with the load. A 20 mm diameter, single-core cable is shown in Figure 2. The cable is made up of nineteen conducting copper wires and consisted of dual layers of insulation.

**Figure 3:**
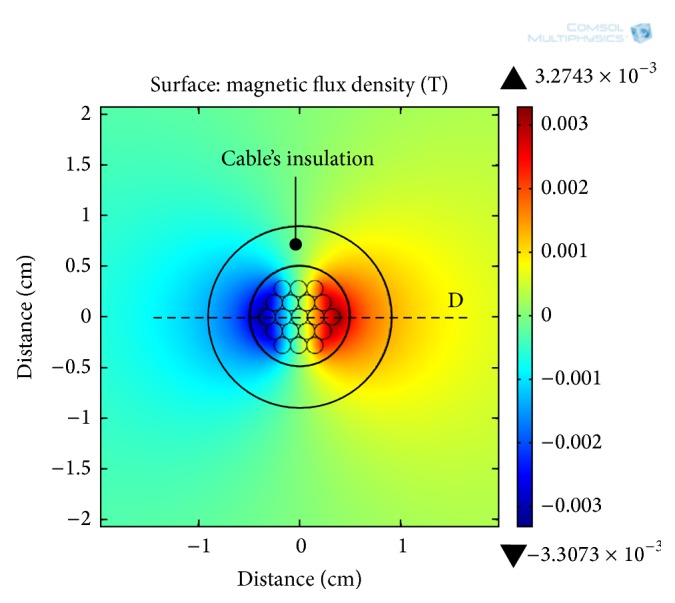
MFD distribution along a cross-section of a single-core insulated cable at a current amplitude of 72 A.

For a cable current of 72 A, a 2D electromagnetic analysis of a single-core insulated cable is carried out in COMSOL Multiphysics and along the cross-section of the cable, the distribution of the MFD around the conductive core is shown in Figure 3. The simulation shows that the MFD produced by the flowing current is intense at a radius of 4 mm from the cable's center. However, this relatively high MFD is unavailable for the energy harvesters, as it resides within the insulation of the cable. For the electrodynamic energy harvesters, the MFD is actually present after the cable's insulation which is after a radial distance of 10 mm from the cable's center.

**Figure 4:**
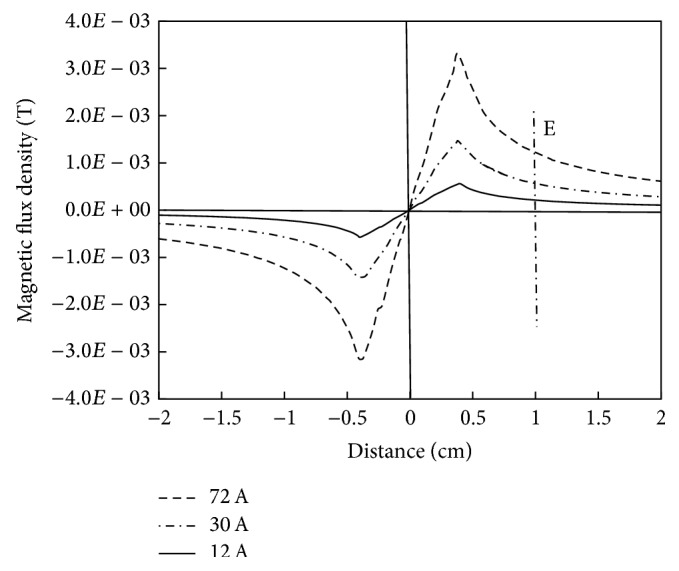
MFD as function of radial distance from the cable's center (line D in Figure 3).

**Figure 5:**
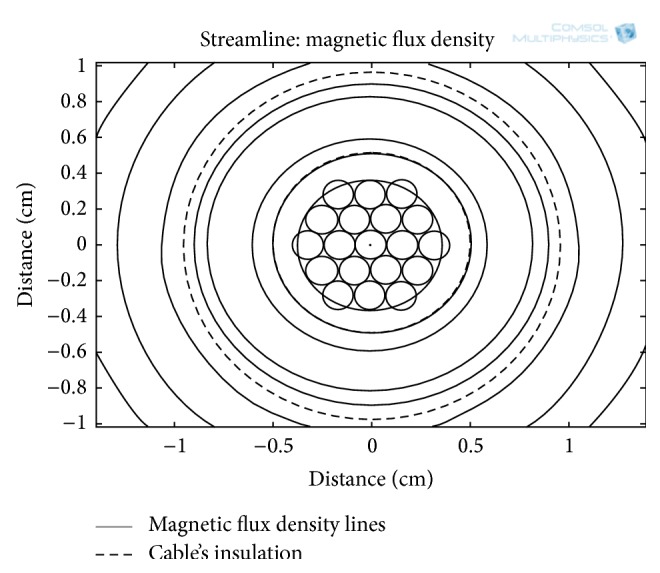
MFD lines along a cross-section of a single-core insulated cable at a current amplitude of 72 A.

For three different current levels (12, 30, and 72 A) in the power line, the simulation results for MFD with respect to radial distance from the center of the power cable are shown in Figure 4. At these current levels, the magnitude of MFD along line D (Figure 3) is obtained and plotted in Figure 4. The MFD is zero at the center of the cable's core; however, moving outward along the radial direction from the cable's center, the MFD increases. At a distance of about 4 mm (line E in Figure 4), the MFD is optimum; however, it reduces drastically when it reaches the outer surface of the cable's insulation (line E in Figure 4). For the energy harvesting, the optimum MFD is inaccessible, since it is inside the cable's insulation. The most feasible location for the electrodynamic energy harvester actually starts from the outer surface of the insulation. At the outer surface of the insulation (10 mm, radial distance), MFD of 0.21, 0.56, and 1.2 mT are available at current amplitudes of 12, 30, and 72 A, respectively. The radial distance from 10 to 15 mm is the most probable position for the external electrodynamic energy harvesters.  Figure 5 shows MFD lines along the cable's cross-section at a current amplitude of 72 A in the power line. All of the MFD lines are concentric with the periphery of the cable's insulation.

**Figure 6:**
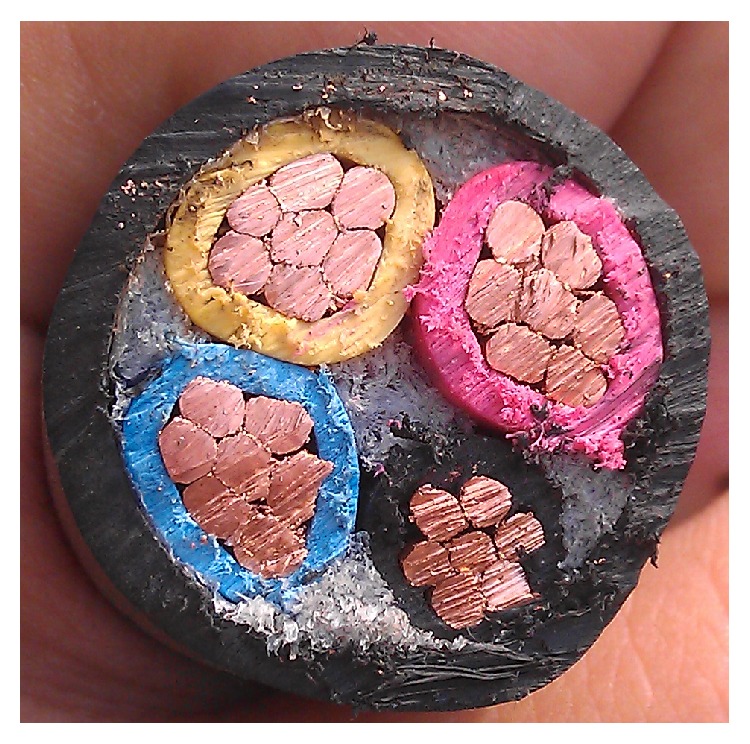
Three-core insulated cable for electrical power transmission.

In three-core cable, each 6 mm diameter core is made up of 7 conducting copper wires and an insulation layer. The three insulated cores are enclosed in a main cable's insulation and the cable's overall diameter is 20 mm, as in Figure 6.

**Figure 7:**
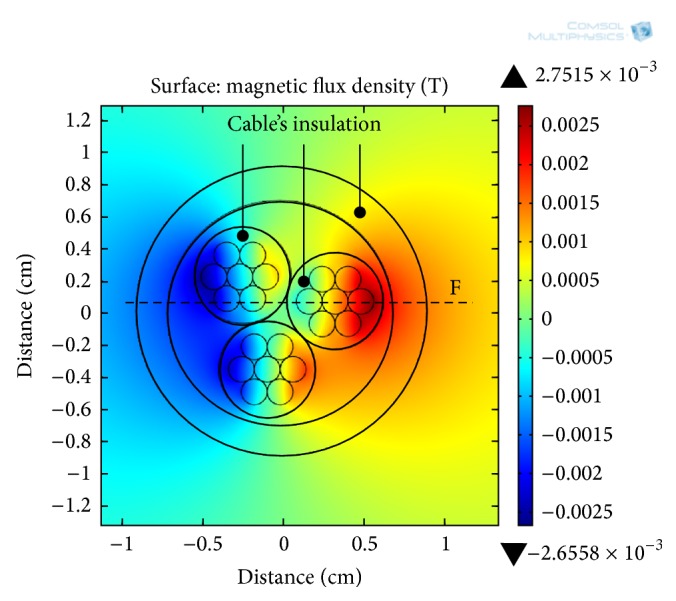
Distribution of a MFD along a cross-section of a three-core cable at a current level of 72 A.

A 2D electromagnetic analysis of a three-core cable is also performed and a MFD surrounding the conductive cores at a cable current of 72 A is depicted in Figure 7. The high MFD distribution (2.75 mT) occurs inside the cable's insulations and near the inner surface of the outer insulation cover (Figure 7), apart from these regions, due to the cancelation of opposite polarity of MFD, the MFD distribution is relatively on lower side.

**Figure 8:**
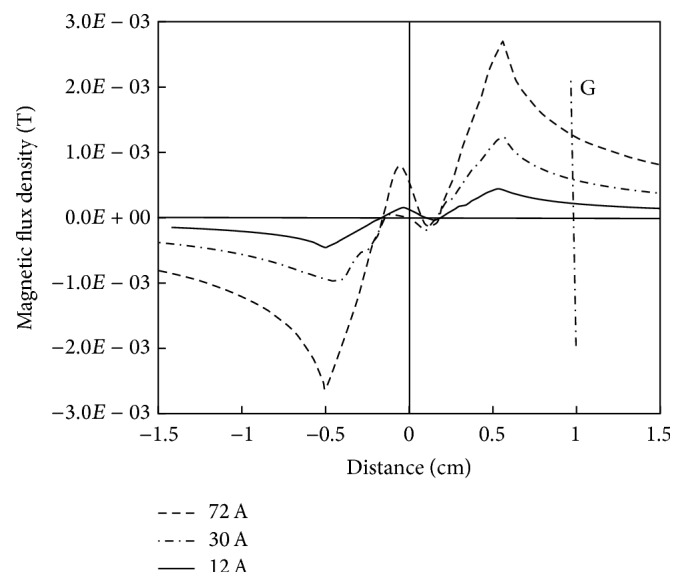
MFD as function of radial distance from the cable's center (line F in Figure 7).

At cable's current levels of 12, 30, and 72 A, the magnitude of MFD (along line F in Figure 7) as function of distance is presented in Figure 8. Near the cable's center, the cable exhibits lower values of the MFD which is attributed to the cancelation of opposite polarity of the MFD at that location. However, due to the superposition of magnetic field lines, the optimum magnetic flux densities of 0.44, 1.23, and 2.7 mT are available inside the cable's insulation (at about radial distance of 5.6 mm). Afterward, the MFD decreases considerably and near the outer surface of the cable (line G in Figure 8), MFD of 0.21, 0.56, and 1.14 mT are available at current levels of 12, 30, and 72 A, respectively, which are approximately the same as in case of simulation of the single-core cable.

**Figure 9:**
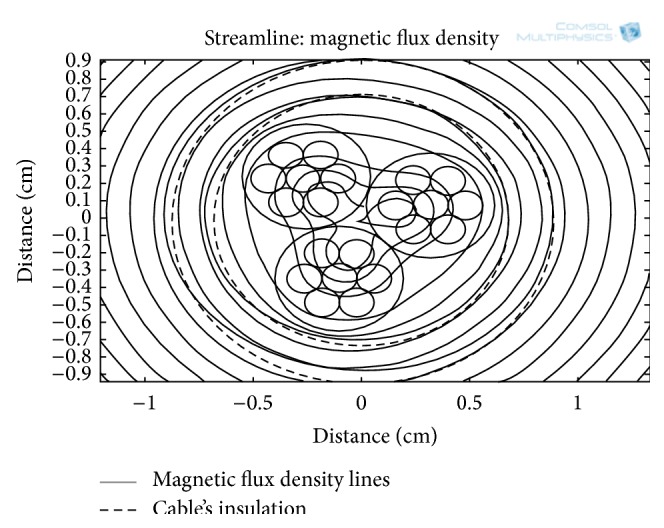
MFD lines along a cross-section of a three-core cable at a current level of 72 A.

Figure 9 shows the MFD lines produced when a current of 72 A is passed in the three-core cable. In a single-core cable (Figure 5), the MFD lines are all circular and concentric with the round periphery of the cable. However, for three-core cable, the MFD lines inside the cable are of three-knobbed (triangular) shape, which then protruded into concentric circles outside the insulation of the cable.

### 3.2. Analytical Modeling for Electrodynamic Energy Harvesters

In the power cable, when the electric current is flowing, the stray magnetic flux density [22, 31]

(1) $$\begin{array}{c} B_{\theta }\left(\;t\;\right)=\frac{\mu I\left(t\right)}{4\pi r} \end{array}$$

produced arround it depends on the cable's current* I*(*t*), the magentic permeability *μ* of the cable's core material, and the radial distance* r* from the centerline of the cable.

The small variation

(2) $$\begin{array}{c} dB_{\theta }\left(\;t\;\right)=\frac{-\mu I\left(t\right)}{4\pi }\frac{1}{r^{2}}dr \end{array}$$

in the magnetic flux density along the radial distance *dr* leads to the equation

(3) $$\begin{array}{c} B_{\theta }\left(\;t\;\right)=\frac{-\mu I\left(t\right)}{4\pi }\overset{r_{2}}{\underset{r_{1}}{\int }}\frac{1}{r^{2}}dr \end{array}$$

for the stray magnetic flux density around the electrical cable in terms of radial distances* r*_1_ (from the center of the cable to the inner side of copper coil or annular plate) and* r*_2_ (from the center of the cable to the outer edge copper coil or annular plate).

#### 3.2.1. Analytical Modeling for Inductive Energy Harvester

By Faraday's law the voltage induced

(4) $$\begin{array}{c} V\left(\;t\;\right)=-N\int \frac{dB_{\theta }\left(t\right)}{dt}dA \end{array}$$

in the coil of the inductive energy harvester can be obtained with the magnetic flux density *B*, coil's individual turn area *A*, and coil's number of turns *N*.

For the coil of the harvester, the loop area

(5) $$\begin{array}{c} A=h\overset{r_{2}}{\underset{r₁}{\int }}dr \end{array}$$

of the coil's single turn can be obtained with the coil's height *h* and radial distances* r*_1_ and* r*_2_.

Substituting (3) and (5) in (4) yields the induced voltage

(6) $$\begin{array}{c} V\left(\;t\;\right)=-N\frac{\mu }{4\pi }h\frac{dI\left(t\right)}{dt}\overset{r_{2}}{\underset{r_{1}}{\iint }}\frac{1}{r^{2}}dr\;dr \end{array}$$

in the harvester's coil.

For the alternating current *I*(*t*) = *I*_*o*_sin⁡(*ωt*) in the power cable, (6) reduces to the voltage generation

(7) Vt−NωIoμ4πhr2−r12r2r1cos⁡ωt=−NωIoμ4πhw2r2r1cos⁡ωt

in terms of the coil's width *w* = *r*_2_ − *r*_1_, frequency *ω*, and amplitude *I*_*o*_ of the cable's current.

#### 3.2.2. Analytical Modeling for Capacitive Energy Harvester

In between the annular parallel plates of the capacitive energy harvester, the curling stray magnetic field density** B** produces the changing electric field** E** that can be obtained from Maxwell's equation [31]

(8) $$\begin{array}{c} \nabla \times B=\mu_{0}\epsilon_{0}\frac{\partial E}{\partial t}, \end{array}$$

where *μ*_0_ and *ϵ*_0_ are magentic permeability and permittivity of free space, respectively.

The curl

(9) $$\begin{array}{c} \nabla \times B=\frac{1}{r}\left[\;\frac{\partial }{\partial r}\left(\;rB_{\theta }\;\right)\;\right]\hat{z} \end{array}$$

of the stray magnetic field density** B** around the cable and (3) yields the equation

(10) ∇×B−μIt4π1r∂∂r∫r1r21rdrz^=−μIt8π1r22−1r12z^

which, when substituted in (8), provides an analytic model for the changing electric field

(11) $$\begin{array}{c} \frac{\partial E}{\partial t}=\frac{-\mu I\left(t\right)}{8\pi \mu_{0}\epsilon_{0}}\left[\;\frac{1}{r_{2}^{2}}-\frac{1}{r_{1}^{2}}\;\right]\hat{z} \end{array}$$

between the plates of the harvester in terms of radial distance* r*_1_ from the center of the cable to the inner side of annular plate and radial distance* r*_2_ from the center of the cable to the outer side of the annular plate.

For alternating current (*I*(*t*) = *I*_*o*_sin⁡(*ωt*)) integration (11) with respective time yields an electric field

(12) $$\begin{array}{c} E=\frac{-\mu }{8\pi \mu_{0}\epsilon_{0}}\left[\;\frac{1}{r_{2}^{2}}-\frac{1}{r_{1}^{2}}\;\right]I_{0}\omega \cos\left(\;\omega t\;\right)\hat{z} \end{array}$$

between the harvester's plates as a function of time, which can be utilized to obtain the induced voltage

(13) $$\begin{array}{c} V=\frac{E}{g}=\frac{-\mu }{8\pi g\mu_{0}\epsilon_{0}}\left[\;\frac{1}{r_{2}^{2}}-\frac{1}{r_{1}^{2}}\;\right]I_{0}\omega \cos\left(\;\omega t\;\right)\hat{z} \end{array}$$

for the capacitive energy harvester as a function of the gap *g* between the annular plates.

## 4. Fabrication of Electrodynamic Energy Harvesters

**Figure 10:**
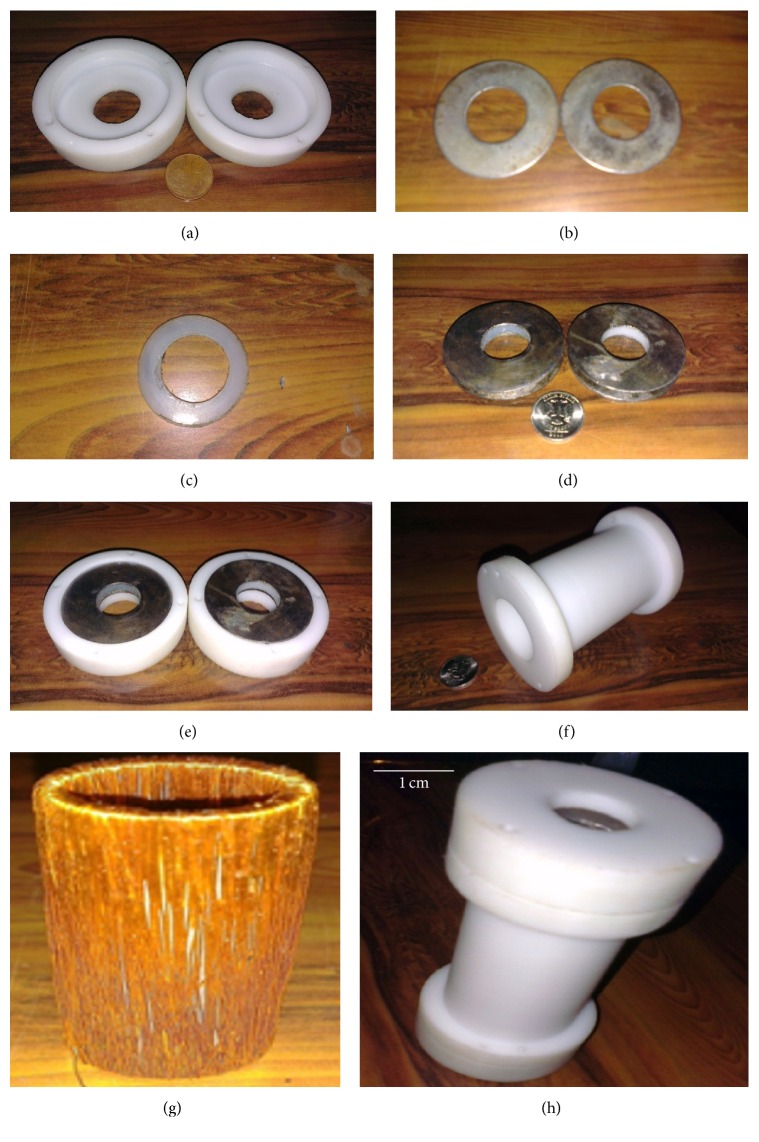
Fabrication of prototype-1: (a) capacitive energy harvester holder caps, (b) annular mild steel conductive plates, (c) Teflon spacer, (d) assembled capacitive energy harvesters, (e) capacitive energy harvesters in Teflon holder caps, (f) Teflon housing for inductive energy harvester, (g) copper wire wound around the cylindrical steel core, and (h) assembled prototype-1.

Five electrodynamic energy harvester prototypes have been fabricated, among which two are hybrid (having combined inductive and capacitive harvesters) and the remaining three are purely inductive energy harvesters. The fabrication of hybrid energy harvester, prototype-1, is shown in Figure 10. The end holder caps for the capacitive portion of the energy harvester are produced from Teflon with conventional machining (Figure 10(a)). In the end holder caps, the central hole is provided for the power cable to pass through it. Two mild steel (MS), annular discs (Figure 10(b)) and a Teflon ring (Figure 10(c)) are bonded together to produce a parallel plate, capacitive energy harvester (Figure 10(d)); an enameled copper wire is wrapped around an MS hollow cylinder to form the inductive portion of the energy harvester (Figure 10(g)). The end holder caps along the capacitive energy harvesters (Figure 10(e)) are then attached to the main Teflon housing (Figure 10(f)) that contains the inductive coil to produce the hybrid electrodymanic energy harvester prototype-1 (Figure 10(h)).

**Figure 11:**
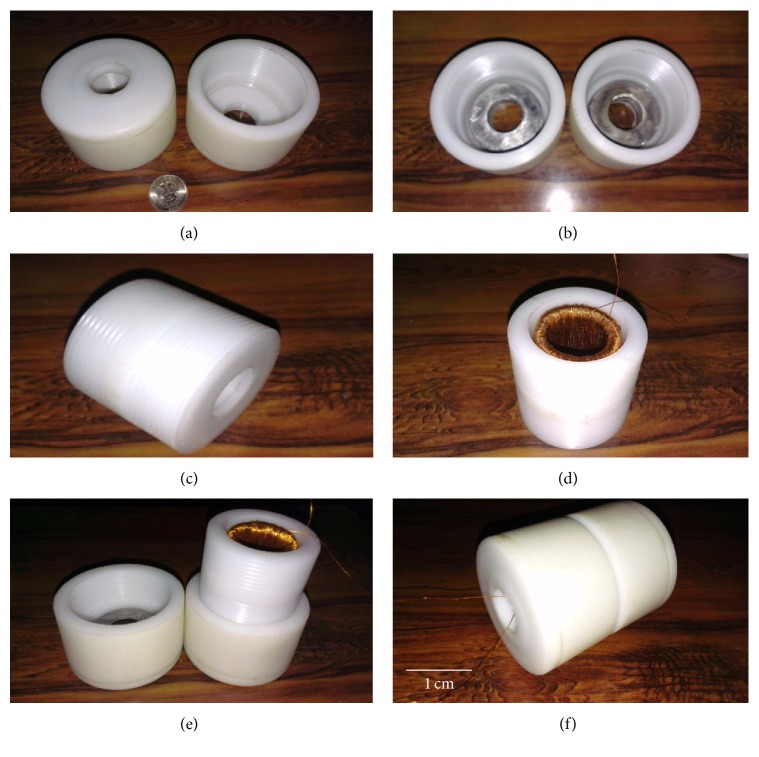
Fabrication of prototype-2: (a) Teflon capacitive energy harvester's holder caps, (b) capacitive energy harvesters placed in screwed holder caps, (c) screwed Teflon housing for inductive energy harvester, (d) inductive energy harvester placed in the main housing, (e) capacitive energy harvester holder cap screwed on the main housing, and (f) assembled prototype-2.

In prototype-2, the height of Teflon holder caps is kept more than that in prototype-1 and internal threads are made (Figures 11(a) and 11(b)). The main housing (Figure 11(c)), which has to accommodate the inductive coil, has external threads (Figure 11(d)). After placing the inductive coil in the main housing, the end holders containing the capacitive energy harvesters are screwed onto the main housing as shown in Figures 11(e) and 11(f).

**Figure 12:**
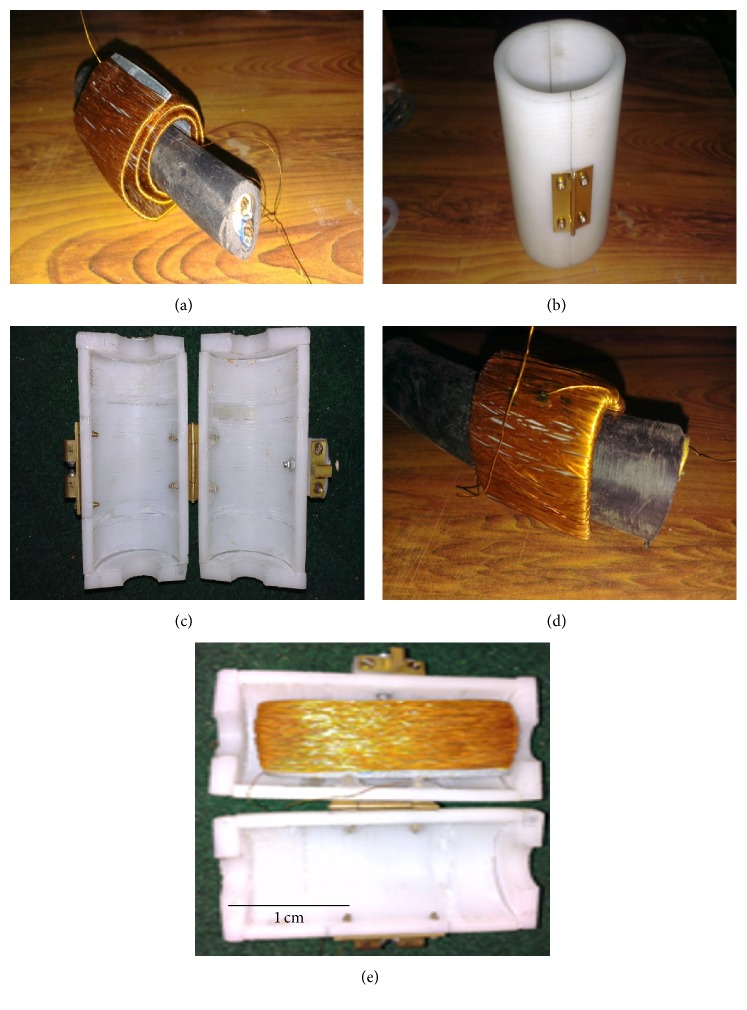
Fabrication of prototype-3 and prototype-4: (a) multiturn inductive core of prototype-3, (b) Teflon split housing hinged, (c) end plates and locking mechanism attached to the split cylinder, (d) wounded steel plate wrapped over a power cable to produce the inductive energy harvester, and (e) inductive energy harvester placed in the split housing prototype-4.

The disadvantage of prototype-1 and prototype-2 is that these cannot be mounted directly upon the in-service power line but rather the power has to be disrupted; the power cable has to be disconnected and is then inserted in the central hole provided in the harvesters. In order to mount the energy harvester on a power cable without disconnecting the power cable, a split design for prototype-3 and prototype-4 is adopted. Prototype-3 and prototype-4 are purely inductive energy harvesters. For energy harvester prototype-3, copper wire is wrapped on a relatively long MS rectangular sheet and then the wound sheet is encircled on top of a power line piece to produce a multiturn inductive energy harvester (Figure 12(a)). A simple Teflon housing is produced by fabricating a hollow cylinder with conventional machining. The hollow cylinder is longitudinally sliced in half and then hinged (Figure 12(b)). Two annular discs are produced which are then cut in half and bonded to the ends of the split cylinder (Figure 12(c)). In the Teflon housing, a locking mechanism is also provided to securely mount the energy harvester and housing on top of the power cable. However, for an inductive harvester, prototype-4, copper wire is wrapped on a relatively shorter MS rectangular sheet and is then firmly wrapped around the power cable piece to develop the harvester (Figure 12(d)).  Figure 12(e) shows prototype-4, when it is placed in the split Teflon housing.

**Figure 13:**
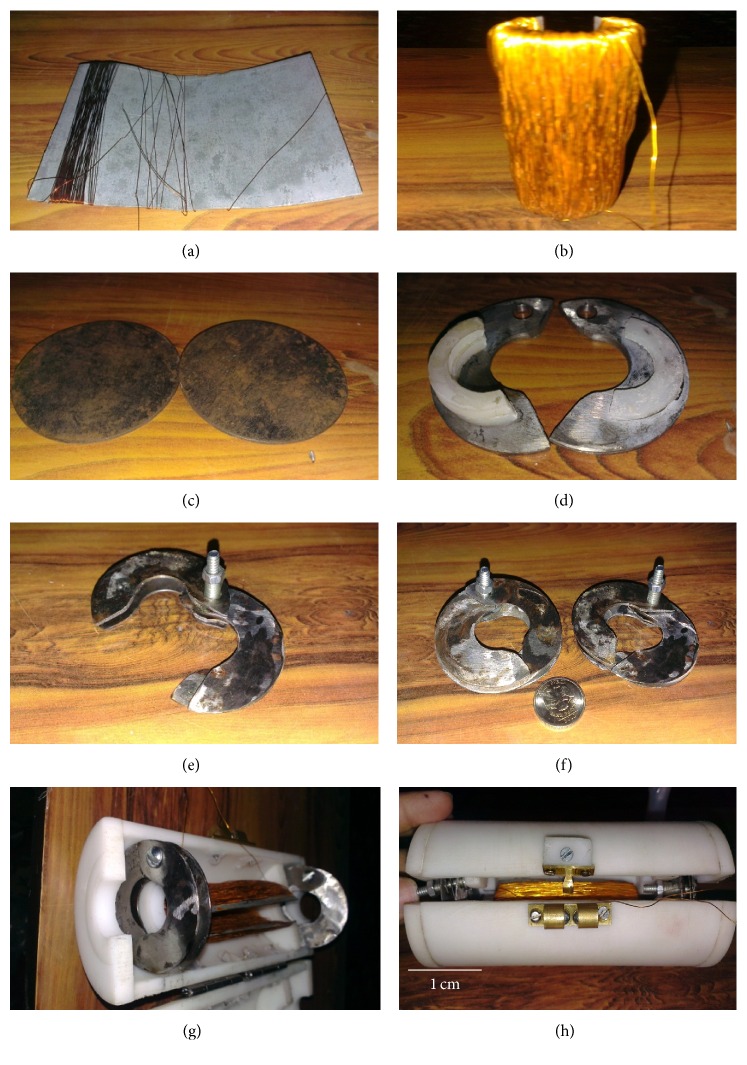
Fabrication of the prototype-5: (a) copper wire wrapping around a mild steel sheet, (b) inductive energy harvester, (c) conductive plates for capacitive energy harvester, (d) Teflon spacer bonded to the split annular plates, (e) assembled capacitive energy harvester in open position, (f) capacitive energy harvesters in closed position, (g) inductor and capacitive energy harvesters placed in split Teflon housing, and (h) assembled prototype-5.

Like prototype-3 and prototype-4, a split design is adopted for the hybrid, electrodynamic prototype-5. The cores of both the inductive and capacitive portions of the prototype-5 are split and can be opened and mounted on the in-service power line. The fabrication steps of the prototype-5 are shown in Figure 13. A copper wire is wrapped around a rectangular piece of MS sheet (Figure 13(a)). The wound MS sheet is then bended on the piece of a power cable to produce a split cylindrical wound coil of the inductive harvester (Figure 13(b)). MS discs are used to produce the split capacitive energy harvesters (Figure 13(c)). In each disc, a central hole and a hole at the side are drilled and then cut in half. Teflon spacers (half ring) are then bonded to each plate (Figure 13(d)) and with small size nut and bolt these are assembled together to produce the split capacitive energy harvester (Figures 13(e) and 13(f)). The inductive energy harvester along the two capacitive energy harvesters is placed in the Teflon split cylindrical housing as shown in Figures 13(g) and 13(h). The developed electrodynamic energy harvesters' dimensions and necessary parameters are listed in Table 2.

## 5. Characterization of Prototypes

**Figure 14:**
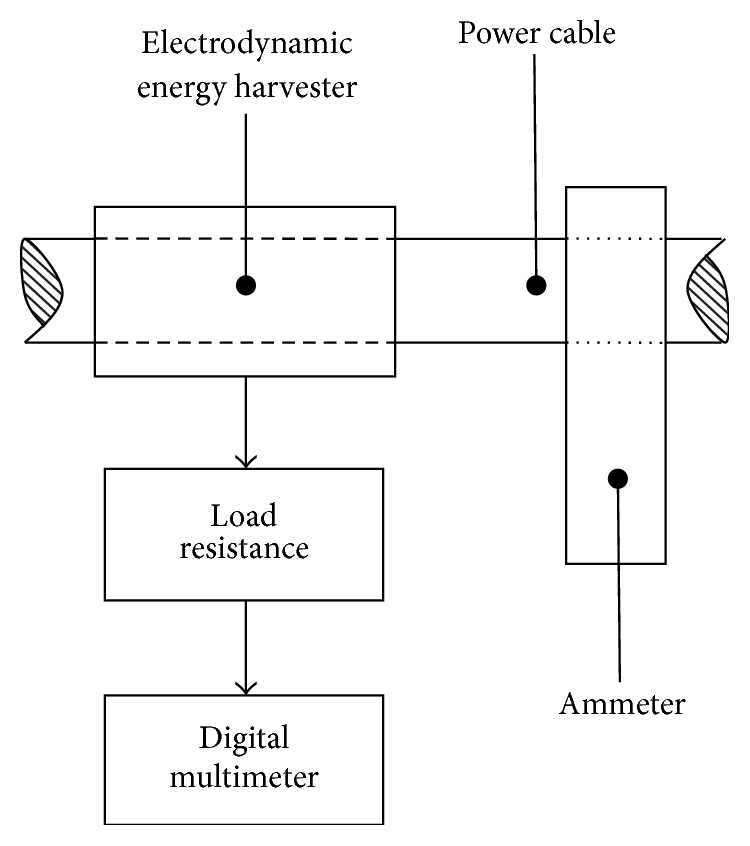
On-spot experimental setup for the characterization of developed energy harvesters.

**Figure 15:**
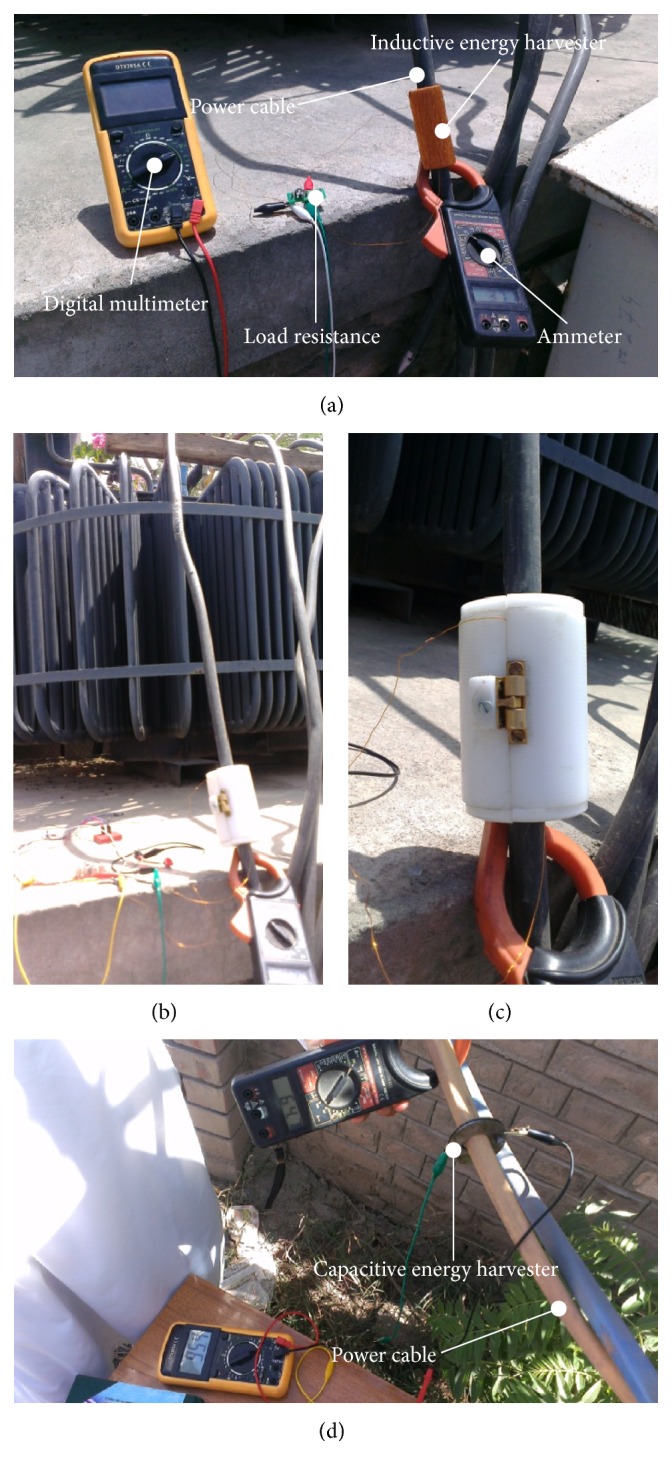
Characterization of developed prototypes: (a) load voltage measurement of inductive energy harvester, (b) inductive energy harvester enclosed inside Teflon housing, (c) close-up view of inductive energy harvester during experimentation, and (d) measurement of voltage from the capacitive harvesting unit.

By using the experimental setup shown in Figure 14, the characterization of the developed electrodynamic energy harvesters is performed. The on-spot experimental setup comprised a clamp-on ammeter for monitoring the electric current through the power cable, a digital multimeter (DMM) for the open circuit and load voltage measurement produced by the installed energy harvester, and a variable load resistance to predict the performance of prototypes under various loading conditions.  Figure 15 shows the experimentation of inductive and capacitive energy harvesters when these are mounted over an insulated 220 V output power cable of a 400 kVA electrical transformer.

Experiments are performed on all the prototypes and data is collected and plotted for both inductive and capacitive harvesters. The induced voltage in the inductive harvester depends on a number of factors, such as number of turns of coil wound around the core, the core size of the inductive harvester, and the current level in the cable which produces the stray magnetic field around the cable. If the number of turns is more, high voltage level will be induced. The same is the case with the size of the harvester, and the cable current is also the main factor which affects the induced voltage. If the current flowing through the cable is greater, the magnetic flux around the coil will be stronger and in turn greater number of magnetic field lines will pass through the coil loop of the inductive harvester and therefore high voltage will be induced.

**Figure 16:**
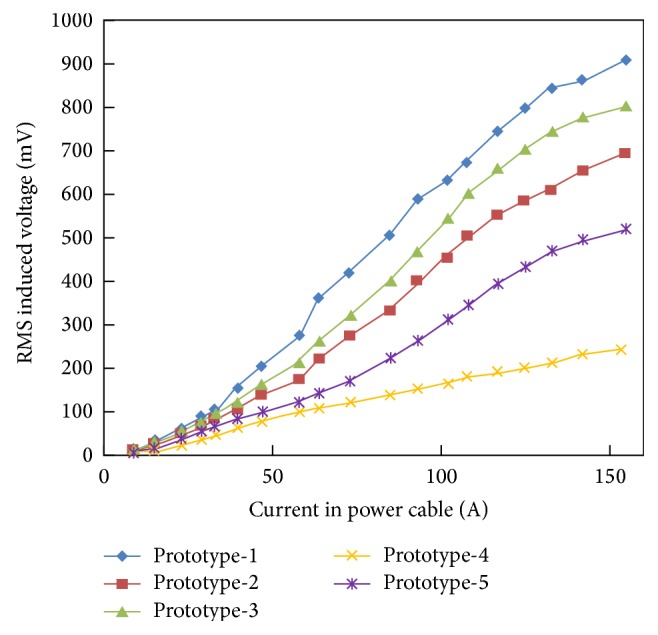
Induced load voltage in inductive energy harvesters as a function of cable current.

Figure 16 shows the induced load voltage generated by the inductive energy harvester prototypes which are mentioned in Table 2. Each prototype is attached to the optimum load (equal to coil's resistance) and the potential drop across the load resistance is measured with DMM. The measurements are taken at different durations during a particular summer day. As a result of the load variation on the transformer, the current drawn from the electrical transformer increases from 8 to 155 A. As the current in the power line is increased, depending upon the harvester's core dimensions and number of coil's turns, the induced voltage accordingly increases in each inductive energy harvester. A maximum load voltage of 908 mV is obtained from prototype-1 at the maximum current level of 155 A.

**Figure 17:**
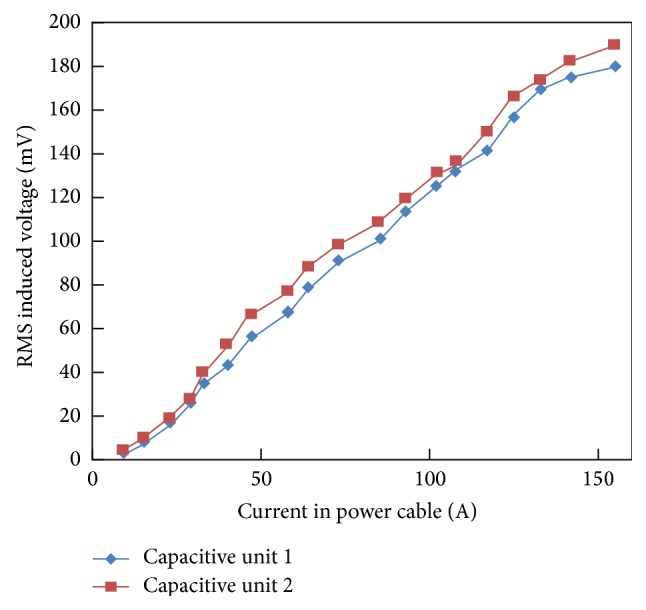
Induced load voltage in capacitive energy harvesters as a function of cable current.

However, when the capacitive energy harvesters are mounted over the power line, the induced voltage as a function of cable current is shown in Figure 17. Likewise, the inductive energy harvester prototypes and the voltage generated by the capacitive harvester prototypes also increase with the increase in the cable current through the power line. Both capacitive energy harvesters produced a maximum voltage of approximately 190 mV at the cable current level of 155 A. The slight variations in the measurements from these capacitive energy harvesters are due to fabrication uncertainties of the harvesters.

**Figure 18:**
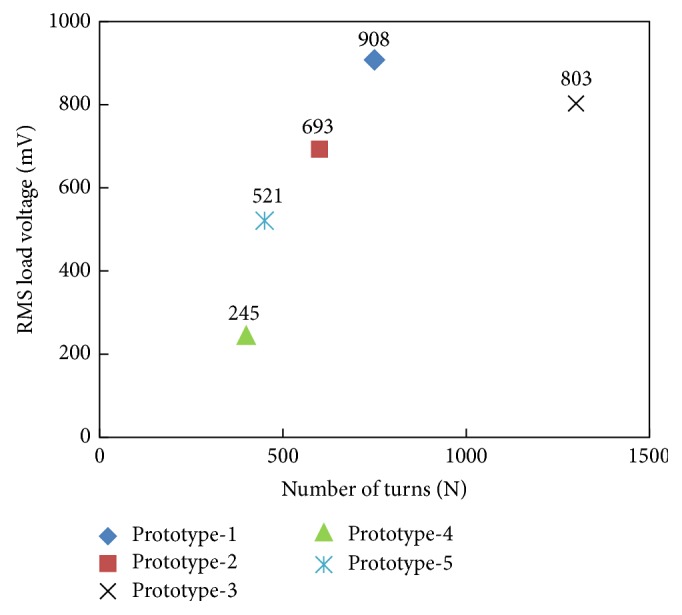
Induced load voltage in inductive energy harvesters as a function of number of coil turns at current level of 155 A.

In inductive energy harvesters, the dependence of induced voltage on numbers of coil's turns is shown in Figure 18. The data is plotted at current amplitude of 155 A in power line. The developed inductive energy harvester prototypes differ from each other with respect to number of turns, core height, and number of core wraps around power cable. Normally, in these energy harvesters, the larger the number of coil's turns, the higher the induced voltage, except prototype-3. In prototype-3, the number of turns is kept 1300 which is comparatively greater than the coil's turns in other prototypes; however, due to core multiwraps around the power line, the distance of wound coil in second and third wraps from the cable's center is relatively more and the stray magnetic field available at these outer wraps is comparatively weaker. In reality, it might be possible that the third wrap is producing either very little voltage or no voltage at all and actually may just act like a load resistance connecting to the other two wraps of the energy harvester and resulting in the reduction of induced voltage.

**Figure 19:**
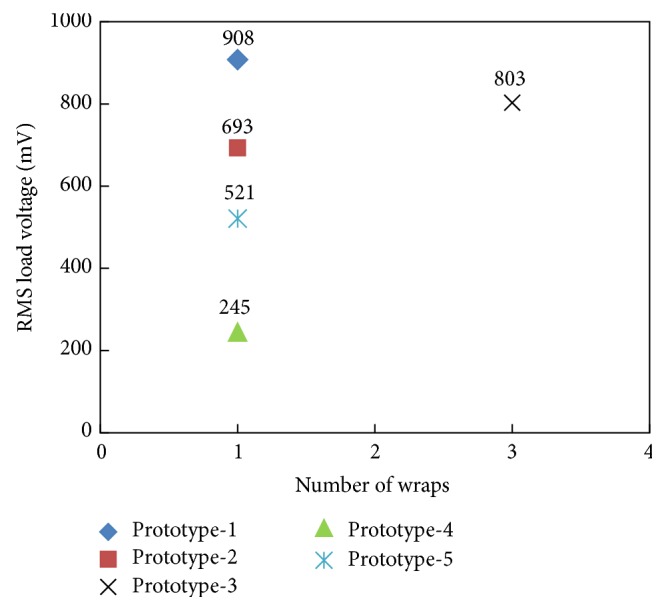
Induced voltage in inductive energy harvesters as a function of number of core's wraps at 155 A current level.

At current level of 155 A in the power line, the induced load voltage as a function of core wraps is shown in Figure 19. Only prototype-3 has three-core wraps; however, the remaining inductive energy harvesters are produced in a single wrap. In single wrap energy harvesters, prototype-1 and prototype-2 due to larger number of coil turns (750 and 600, resp.) and having larger core heights (9 cm and 4.5 cm, resp.) are generating relatively higher voltage levels (908 and 693 mV, resp.). However, prototype-3, in which the core height is 3.5 cm (less than that in prototype-1 and prototype-2), is producing 803 mV voltage which is better than all the prototypes except prototype-1. With lesser core height, the better voltage generation by prototype-3 is attributed to the multiwrap nature of the harvester's core.

**Figure 20:**
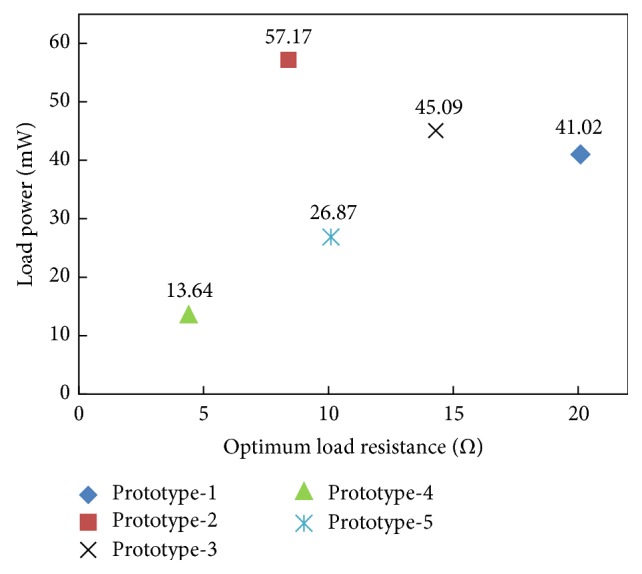
Load power in inductive energy harvesters as a function of optimum load resistance at 155 A.

Optimum values of load power when an optimum load resistance (equivalent to the harvester's coil resistance) is attached to the inductive harvesters at a cable current level of 155 A are shown in Figure 20. Although maximum load voltage is generated by prototype-1 (Figure 19), due to lower coil's resistance of prototype-2 and prototype-3 (which is 7.5 Ω and 14.3 Ω), comparatively, larger power levels of 57.17 mW and 45.09 mW are produced, respectively, by prototype-2 and prototype-3.

**Figure 21:**
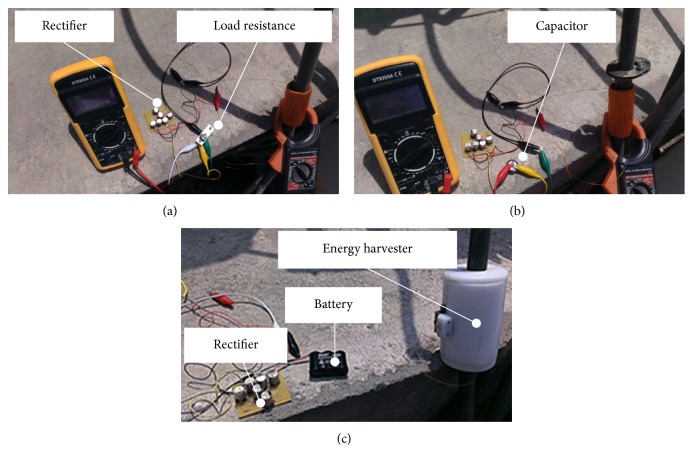
Photographs during voltage rectification and charging: (a) inductive energy harvester connected to a rectifier, (b) charging of a super capacitor with a harvester's rectified voltage, and (c) charging of a rechargeable battery with a harvester's rectified voltage.

The output voltage of the electrodynamic energy harvester is alternating; however, a wireless sensor node requires a DC voltage for its operation. In order to transform the AC voltage of the harvester into DC voltage, the energy harvester ptototype-5, is integrated with an AC to DC rectifier as shown in Figure 21(a). The rectified voltage is then used to charge a super capacitor (1 F, 5.5 V) (Figure 21(b)) and a Nickel-Cadmium (3.8 V) rechargeable battery (Figure 21(c)).

**Figure 22:**
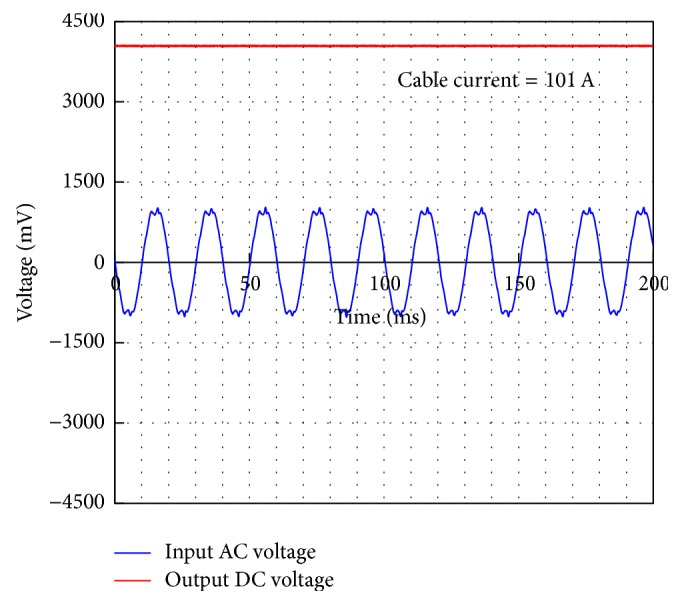
AC and DC voltage levels produced by the inductive energy harvester, prototype-5 at a cable current level of 101 A.

The harvester's AC output voltage (before the rectifier) and the rectified DC voltage as a function of time are depicted in Figure 22. The NI DAQ card is utilized to acquire the signals. When the rectifier is attached to the energy harvester, the optimum load condition changes to 180 Ω. An optimum load resistance of 180 Ω is connected to the output terminals of the rectifier and the measurement is recorded for a cable current level of 101 A. Under these conditions, the harvester's produced an AC voltage with amplitude of 1020 mV, which is converted into 4058 mV DC voltage with the rectifier.

**Figure 23:**
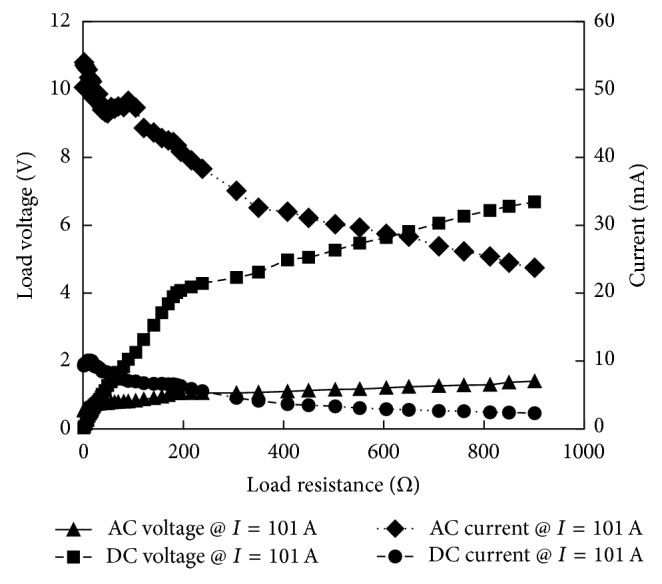
Load voltage and current before and after rectification when the prototype-5 is operating at a cable current level of 101 A.

**Figure 24:**
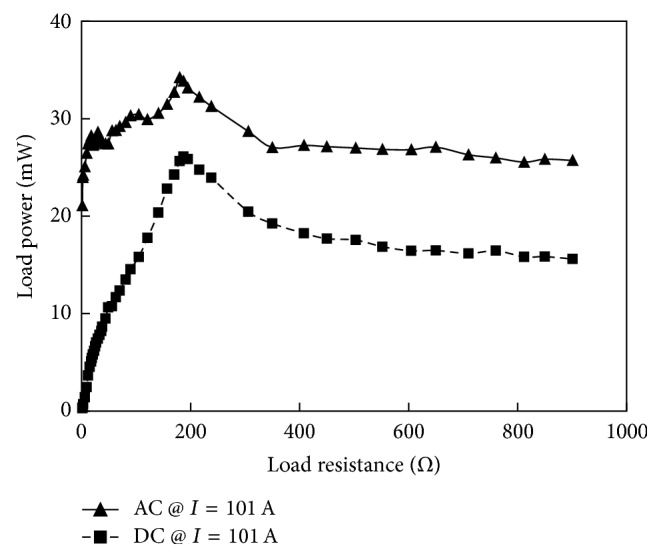
Load power before and after rectification when the prototype-5 is operating at a cable current level of 101 A.

Figure 23 shows the dependence of harvester's AC output (rms) and the DC output (produced after rectification) on the load resistance which is connected to the rectifier. Both the voltage and current measurements are taken under the cable current level of 101 A and are utilized to obtain the power levels (Figure 24) of the system before and after the rectification. Due to the rectifying circuit voltage transformation factor that is greater than 1, the DC load voltage levels are larger than the corresponding rms AC voltage levels. However, the harvester's AC current levels are relatively on the higher side in comparison to the corresponding DC current levels at the same load.

Load power levels produced by protyotype-5 as a function of load resistance attached to the rectifier are shown in Figure 24. When the rectifying circuit is integrated to the prototype the optimum load condition changes from 10.1 Ω to 180 Ω. The difference in the generated and rectified power levels is the power utilization of the rectifier. Under optimum load (180 Ω), the energy harvester generated 34.2 mW power which after rectification is reduced to 26.1 mW.

**Figure 25:**
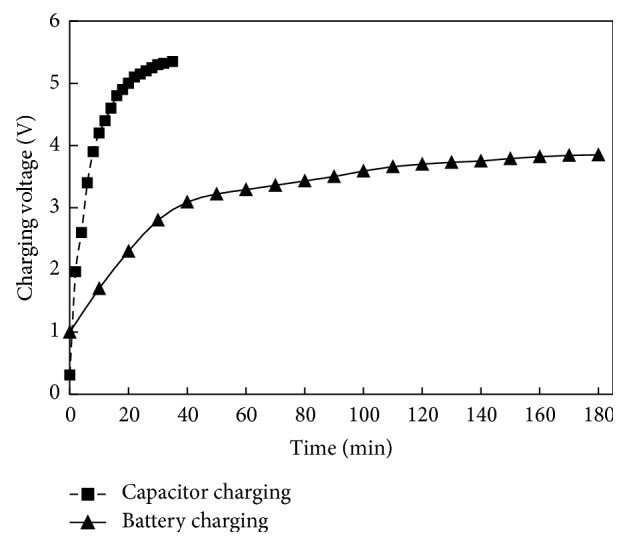
Super capacitor's and battery's voltage with respect to charging time.

Figure 25 shows the charging of a super capacitor (1 F, 5.5 V) and a Nickel-Cadmium (3.8 V) rechargeable battery as function of time. With the rectifier, the output voltage of the prototype-5 is first converted to DC voltage and is then utilized to charge the super capacitor and battery. When the output of the rectifier is supplied to the super capacitor, in the beginning it charges rapidly; however, after 10 minutes, the charging process considerably slowed down. It took 45 minutes for the harvester to charge the capacitor up to the voltage of 5.5 V. However, from the rectifier when the DC voltage is supplied to the battery that has an initial voltage of 1 V, it started charging relatively quicker during the first 40 minutes, as shown in Figure 25. As the charge accumulates in the battery the charging process slowed down. As shown, with the developed harvester the battery is completely charged to an optimum voltage of 3.85 V in 180 minutes.

For temperature monitoring of a 400 kVA electrical transformer, a WTSN is produced and incorporated with the inductive energy harvester prototype-5. The WTSN developed for the monitoring is comprised of a temperature sensor (LM-35, Taxas Instruments Inc., Dallas, USA), transceiver (nRF24L01, Nordic Semiconductor, Oslo, Norway) and an Arduino mini microcontroller (Arduino Pro Mini, ATmega168, Atmel®, California, USA), Ni-Cd rechargeable battery (MP-301, Guangzhou L&U Electronic Co., Ltd., China), and a self-made, step-up AC to DC rectifier.  Table 3 shows different components of the WTSN used in this work.

**Figure 26:**
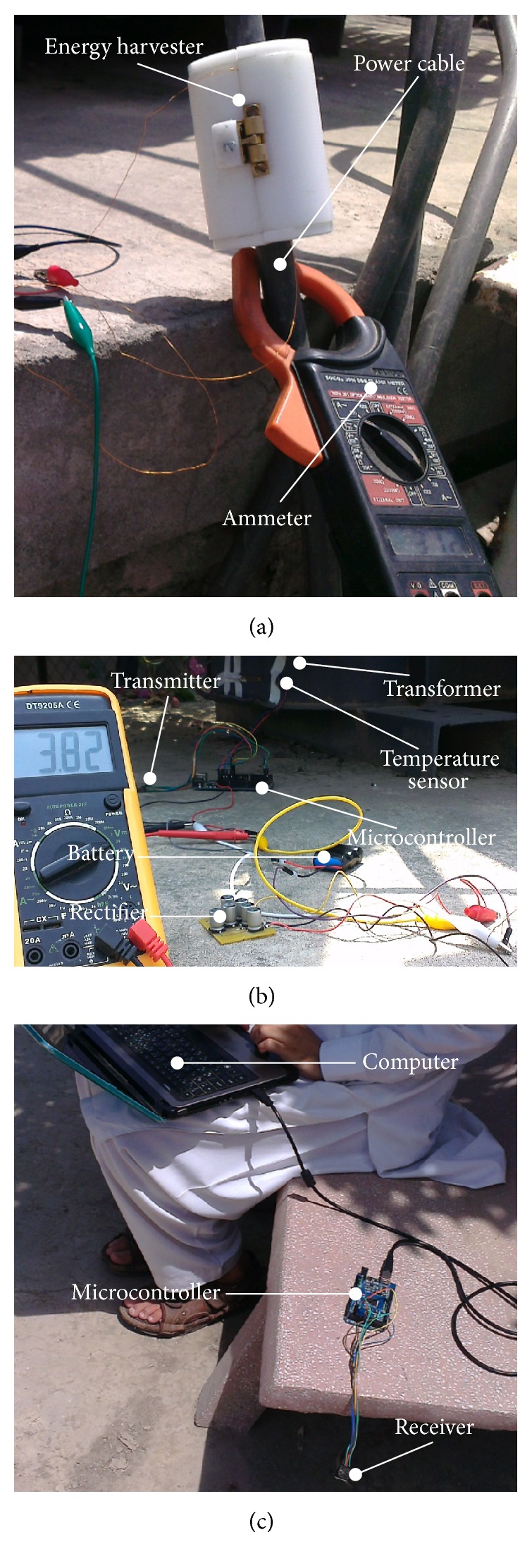
Photographs during wireless temperature monitoring of electrical transformer: (a) developed energy harvester mounted over output power line, (b) rectifier, rechargeable battery, and wireless sensor module integrated with the energy harvester, and (c) receiver connected with notebook computer through microcontroller.

The temperature monitoring system adopted for the 400 kVA electrical transformer is shown in Figure 26. During transmission mode, the WTSN consumed about 108 mW power; however, the optimum power production of prototype-5 is only 26.1 mW. Therefore for the smooth operation of the WTSN, a Ni-Cd battery previously charged with prototype-1 is utilized. With this charged battery, the WTSN is continuously operated for almost 24 hours; however, when the voltage of the battery reduced to 3.3 V, the data reception to the receiver is stopped. With prototype-1, the battery is again completely charged back to 3.8 V in 40 minutes. The charging time (40 minutes) in the monitoring system can be accounted with a break or sleeping time of the system. The sleep mode of the monitoring system could be either kept full for 40 minutes after 24-hour operation or managed in small interval of times (say 1-minute sleep mode after every 30 minutes of WTSN operation).

## 6. Conclusion

For harvesting the stray electromagnetic field around a power line, electrodynamic energy harvesters were developed and characterized in this work. Five inductive energy harvester prototypes and two capacitive prototypes were tested over various current levels in an insulated output power cable of a 400 kVA electrical transformer. Among the inductive energy harvesters, the split-core, prototype-5 produced the maximum rms voltage of 908 mV at 155 A cable current level. However, at the same current level in the power cable, the capacitive energy harvesters generated an rms voltage of 180 mV. AC output voltage of prototype-5 is converted into a DC voltage with a rectifier and a super capacitor and a rechargeable battery is successfully charged. Due to better performance, prototype-5 is also integrated with a wireless temperature sensor node (WTSN) to monitor the temperature of an electrical transformer. Due to lack of moving or mechanical parts, the devised energy harvesters can be utilized for much longer times span. Moreover, the developed electrodynamic energy harvesters can be easily integrated with autonomous sensors for the temperature and electrical current monitoring in smart grids and intelligent transmission lines. Furthermore, the implementation of a thicker core made of ferromagnetic material, such as iron, can highly enhance the voltage and power production in inductive energy harvesters.

## Figures and Tables

**Table 1:** Power consumption data for commercial wireless temperature monitoring sensors.

Sensor node type	Temp. range (°C)	Distance range (m)	Operating voltage (V)	Current		Power consumption (mW)	Ref.
				Transmission mode (mA)	Sleep mode (*µ*A)		
ZigBee, DS18B20, AT89S8253	−55 to +125	10	5.5	50	18	275	[8]
NRF24L01, DS18B20, Arduino UNO	−55 to +125	90	4	30	—	120	[9]
LM-35, AT80C51, Zig Bee	−55 to 150	10	5	50	10	155	[10]
LM35, Arduino UNO, Arduino GSM shield	−55 to 150	GPRS network	5.5	1100	—	6050	[11]
Sensaphone remote monitoring IMS4210	0 to 46	76	6	1300	—	7800	[12]

**Table 2:** Dimension and parameters of developed energy harvester prototypes.

Harvester's type	Description	Prototype-1	Prototype-2	Prototype-3	Prototype-4	Prototype-5
Inductive energy harvester	Material	Mild steel	Mild steel	Mild steel	Mild steel	Mild steel
	Core's height	9 cm	4.5 cm	3.5 cm	3.5 cm	9 cm
	Core's sheet length	6.9 cm	6.9 cm	20 cm	6.9 cm	6.9 cm
	Number of turns of copper coil	750	600	1300	400	450
	Coil's resistance	20.1	7.5	14.3	4.4	10.1

Capacitive energy harvester	Spacer material	Teflon	Teflon	—	—	Teflon
	Outer diameter	5 cm	5 cm	—	—	5 cm
	Inner diameter	2 cm	2 cm	—	—	2 cm
	Thickness	0.7 cm	0.7 cm	—	—	0.6 cm

**Table 3:** Components of devised WTSN for transformer's temperature monitoring.

Component	Model	Main features
Transmitter	nRF24L01	40 m range
Temperature sensor	LM-35	−55 to 150°C temperature range
Microcontroller	Arduino Pro Mini	ATmega168, 3.3 V–5 V, 40 mA, 8 Mhz clock speed
Rectifier	3-step Cockcroft-Walton	AC to DC, step-up voltage
Battery	Ni-Cd rechargeable battery	3.8 V, 450 mAh
